# PTMVision: An Interactive Visualization Webserver for Post-translational Modifications of Proteins

**DOI:** 10.1021/acs.jproteome.4c00679

**Published:** 2025-01-08

**Authors:** Simon Hackl, Caroline Jachmann, Mathias Witte Paz, Theresa Anisja Harbig, Lennart Martens, Kay Nieselt

**Affiliations:** † Institute for Bioinformatics and Medical Informatics (IBMI), University of Tuebingen, Sand 14, 72076 Tubingen, Germany; ‡ VIB-UGent Center for Medical Biotechnology, VIB, Suzanne Tassierstraat 1, Ghent 9052, Belgium; § Department of Biomolecular Medicine, Ghent University, Technologiepark-Zwijnaarde 75, Ghent 9052, Belgium

**Keywords:** post-translational modifications, open search, visualization, Web server

## Abstract

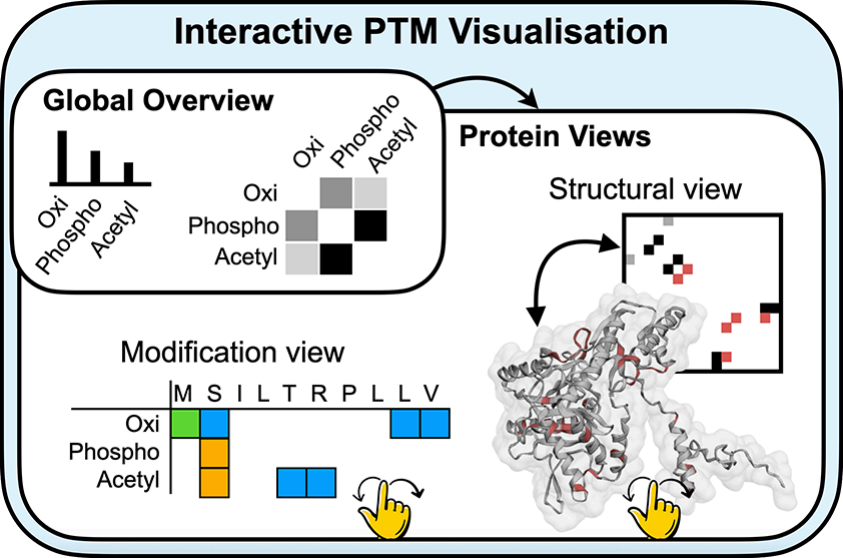

Recent improvements in methods and instruments used in mass spectrometry have greatly enhanced the detection of protein post-translational modifications (PTMs). On the computational side, the adoption of open modification search strategies now allows for the identification of a wide variety of PTMs, potentially revealing hundreds to thousands of distinct modifications in biological samples. While the observable part of the proteome is continuously growing, the visualization and interpretation of this vast amount of data in a comprehensive fashion is not yet possible. There is a clear need for methods to easily investigate the PTM landscape and to thoroughly examine modifications on proteins of interest from acquired mass spectrometry data. We present PTMVision, a web server providing an intuitive and simple way to interactively explore PTMs identified in mass spectrometry-based proteomics experiments and to analyze the modification sites of proteins within relevant context. It offers a variety of tools to visualize the PTM landscape from different angles and at different levels, such as 3D structures and contact maps, UniMod classification summaries, and site specific overviews. The web server’s user-friendly interface ensures accessibility across diverse scientific backgrounds. PTMVision is available at https://ptmvision-tuevis.cs.uni-tuebingen.de/.

## Introduction

Post-translational modifications (PTMs) are chemical modifications that occur after the translation of a protein. PTMs are known to play key roles in a variety of biological processes such as signal transduction,^1^ protein degradation^2^ and transcription activation.^3^ The study of PTMs is therefore essential for understanding how cells regulate these complex processes, and thus how they function. PTMs are typically identified through approaches that require prior selection of the modifications of interest. A common method for isolating and identifying specific types of PTMs from protein samples involves liquid chromatography-tandem mass spectrometry (LC-MS/MS) combined with specific enrichment protocols. For example, phosphorylated peptides can be enriched using immobilized metal affinity chromatography (IMAC)^4^ or titanium dioxide^5^ prior to LC-MS/MS. An even more targeted approach is the use of site-specific antibodies^6^ in combination with Western blotting and immunofluorescence protocols.

In contrast, recent algorithmic and computational advances have enabled the untargeted identification of PTMs from MS experiments,^7−11^ leading to the discovery of numerous new modification sites.^12^ This broadening of the PTM spectrum necessitates more versatile visualization tools to adequately map the ever-increasing complexity observed in these modifications. Currently, PTMs are often visualized using lollipop plots, which are employed by Scop3P,^12^ PhosphoSite Plus,^13^ and FAT-PTM^14^ to show modification sites along the amino acid sequence. AlphaMap^15^ and qPTM^16^ use visualizations that combine the primary structure with stacked bar charts to display PTMs, and annotation tracks showing additional information such as surface accessibility and domains. Furthermore, PeptideAtlas^17,18^ utilizes bar charts to display peptide-spectrum match (PSM) quantities and localization probabilities. Plant PTM Viewer^19^ and iPTMnet^20^ focus on highlighting PTMs directly on the primary structure, while 3D structures have been used in e.g. Bludau et al., 2022.^21^ These diverse techniques collectively facilitate a multidimensional understanding of PTMs, but do not scale to the high-dimensional data derived from open searches, either because these approaches are restricted to only one, or at best a few, modification types, or because the vast amount of PTM types and sites would lead to cluttered figures.

In order to make the large-scale analysis of PTMs more accessible, it is therefore necessary to adapt current and develop new visualization approaches to cope with the new challenges posed by open search results, and to ensure that these visualizations will be easily available for researchers to use on their own results. To address this emerging issue, we have developed PTMVision, a web server that simplifies the visualization of PTM identifications from open searches with a user-friendly interface and interactive, clear visualizations. It provides various visual summaries of mass shifts and modification types at the sample level as well as detailed views at the protein level for both sequence and 3D structure. Designed in close collaboration with proteomics experts, and initiated as an entry to the Bio+MedVis Challenge at the IEEE VIS conference in 2022, PTMVision is accessible to laboratory researchers without requiring coding skills.

## Materials and Methods

PTMVision was implemented as an interactive web application for visualizing post translational modifications (PTMs) from proteomics data: The application implements a Python micro web framework (Flask, v.2.3.3) to manage server-side processing. The front end for clients leverages JavaScript and HTML/CSS to provide a dynamic and responsive user interface. The full application is deployed through containerization with Docker.

The overall user-interface workflow, as well as the individual visualizations were implemented on the basis of Ben Shneiderman’s mantra *overview first, details on demand*.^22^ All visualizations were designed and implemented with the declarative JavaScript framework Apache ECharts^23^ and, for the representation of molecular data, the JavaScript library 3Dmol.js.^24^

To accommodate a broad spectrum of the community, PTMVision was intended to support input formats from various popular proteomics search engines. In order to test this thoroughly and to demonstrate the applicability of PTMVision, input data sets were compiled based either on publicly available LC-MS/MS raw files, which were then processed by the authors of this manuscript, or on published results from proteomics search engines. These include four data sets from PRIDE^25^ (PXD000498,^26^ PXD007740,^27^ PXD025088,^28^ PXD001077^29^), one data set from MassIVE (MSV000091214^30,31^), and one data set made available in a plain CSV format as part of the Bio+MedVis Challenge at the IEEE VIS conference in 2022.

This collection of data sets was analyzed using ionbot,^10^ MSFragger,^7,32^ Sage,^33^ MaxQuant,^34^ Spectronaut,^35^ and MS-GF+.^36^ An explicit assignment of which data set was analyzed with which proteomics search engine, the exact input and output files, as well as a description of the projects can be found in Supplementary Table S1. All seven files with PTM site information (one for each of the search engines used plus the plain CSV format file) were processed with PTMVision and can be accessed as example sessions.

## Results

### PTMVision Web Server

In this section we present PTMVision and its key features to visualize and analyze protein PTM data. Currently, PTMVision directly supports parsing of PTM sites from the output of ionbot,^10^ MSFragger,^7^ MaxQuant,^34^ Sage,^33^ Spectronaut,^35^ from files formatted in the mzIdentML standard,^37^ as well as all PSM and peptide formats that can be handled by the psm_utils API.^38^ Finally, PTMVision also supports PTM site information uploaded as a user-compiled comma separated values (CSV) file containing the UniProt^39^ protein IDs, modification sites, and the modification names. Extensive information on this format can be obtained directly from the PTMVision Web site. Furthermore, processed data can be downloaded in binary format (.zlib) to allow a session to be relaunched easily.

**Figure 1:**
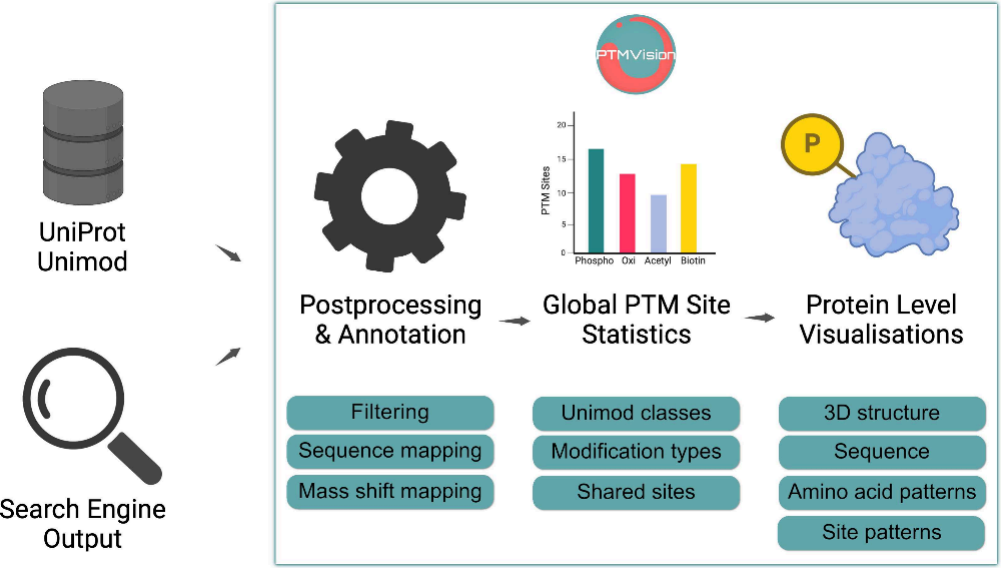
*PTMVision workflow*. PTMVision is a web server for the interactive visualization of PTM site information parsed from output of open or closed searches. Postprocessing includes false discovery rate filtering, protein annotation with UniProt, mapping of modification sites on the protein sequence, and mapping mass shifts to UniMod modifications. Users can explore the data interactively on sample level (site counts, shared sites, mass shift distributions, UniMod modification classes), and on protein level. Protein level information is presented both in primary sequence context and in 3D structural context.

In terms of data processing, the provided input is first parsed and, if available, filtered on identification confidence scores, such as the false discovery rate (FDR), before extracting PTM site information (Figure 1). Afterward, identified proteins are mapped to their corresponding UniProtKB^39^ entries to retrieve their sequences and annotations, while PTMs and/or mass shifts are mapped to UniMod^40^ modifications. The last part of the processing pipeline includes the calculation of basic summary statistics of modified sites for the entire data set, and their visualization in the overview panel. These summary statistics include shared sites between PTM types, and mapped UniMod classification distributions.

In the next step, the user can select a protein either by name or by UniProt accession, or by filtering for specific modifications. The PTM sites of the selected protein can then be investigated with interactive visualizations, such as presence/absence plots, contact maps, and 3D structures, annotated with current knowledge provided by UniProt. To aid optimal interpretation, each visualization is described in a user guide available on the PTMVision Web site.

### Postprocessing

Depending on the search engine, different postprocessing steps are performed. Parsing is done using psm_utils^38^ along with custom Python scripts. PSMs are filtered at 1% FDR, and decoy matches and peptides mapping to multiple proteins are removed. The position of each modification is mapped to the protein sequences retrieved from UniProt,^39^ and predicted protein structures are retrieved from the AlphaFold Protein Structure Database.^41^ Mappings from mass shifts and UniMod^40^ names to UniMod identifiers and classifications are done with Pyteomics^42^ at a user defined mass tolerance (default: 0.001 Da). If a mass shift matches to multiple or no UniMod modifications, the mass shift is used as the label in the visualizations. For FragPipe/MSFragger,^7,32,43−45^ PSMs are removed when the assigned mass shifts are explained by a combination of modifications or for which more than one possible localization was reported. If the user is not interested in the analysis or visualization of specific modification classes defined by UniMod, these can be excluded from the analysis.

### Summary Visualizations at Sample Level

**Figure 2:**
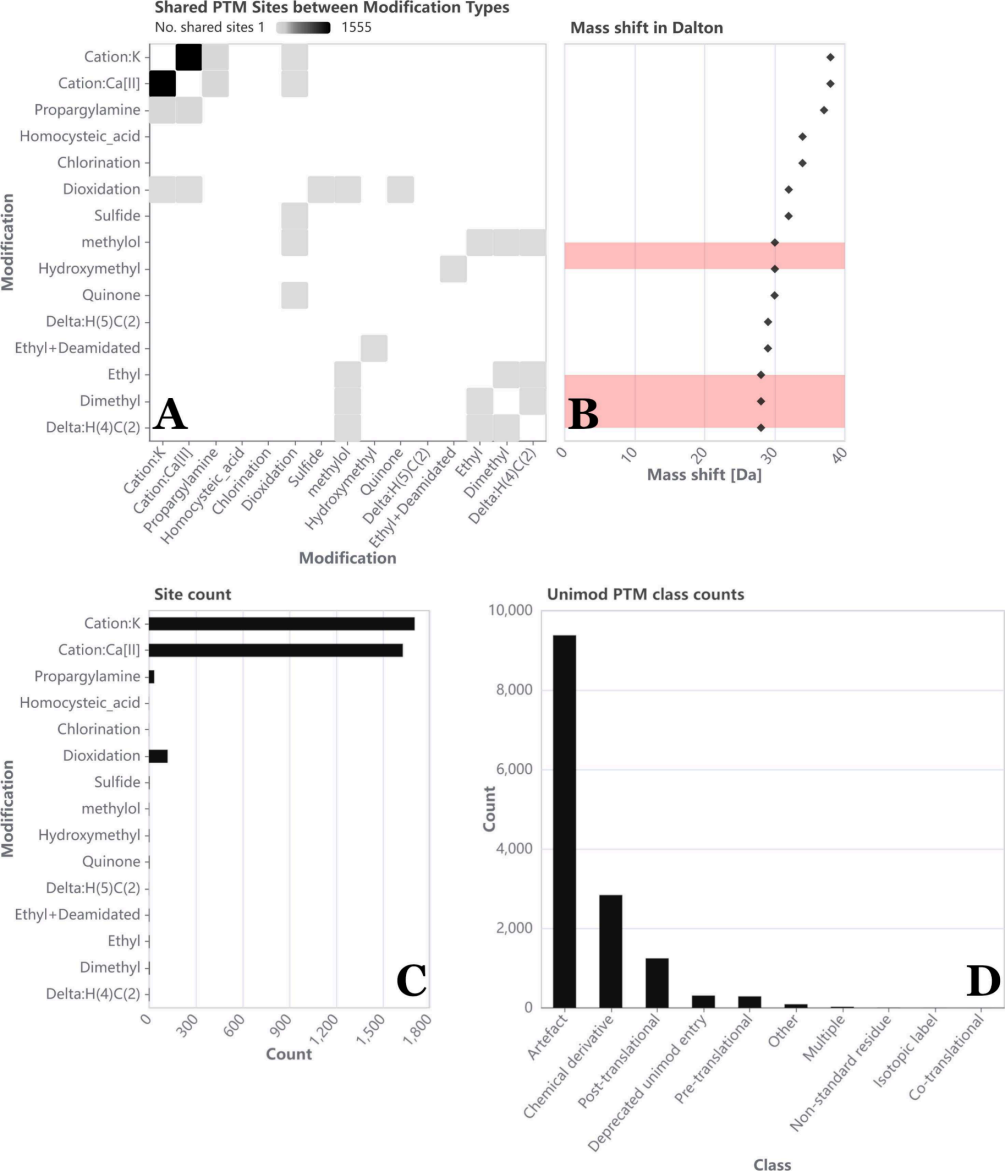
*Sample-level visualizations of PTMVision*. All subfigures are zoomed in and therefore do not reflect all observed modifications of the data. (A) Number of shared sites between modifications (e.g., sites that were both observed with sulfide and dioxidation modifications) visualized as a heatmap plotting all types against all types; cell shade indicates the number of shared sites. (B) A mass shift scatter plot and (C) an absolute modification count bar chart are plotted with a shared *y*-axis. The Modification axis can be sorted according to either mass shift or number of sites. When sorted by mass shift, modification type pairs that have a mass shift difference smaller than a user defined threshold, and are therefore hard to distinguish via MS, are flagged in red in (B). (D) Bar chart showing the distribution of assigned UniMod classes across all modifications, e.g. Artefact (accidentally induced during sample preparation), Chemical derivative (intentional modifications for experimental purposes), or Post-translational (*in vivo* PTMs).

PTMVision provides sample-level statistics for a quick overview of the PTM site composition of the sample (see Figure 2). These visualizations are designed to answer the following questions: Which modifications were identified, and at how many sites (Figure 2 C)? Which modifications share a lot of sites (Figure 2 A)? Which mass shifts do the different modifications have, and which modifications might be wrongly assigned due to multiple options with similar masses (Figure 2 B)? How many modifications were accidentally induced during sample preparation, how many are intentional modifications for experimental purposes, how many are *in vivo* modifications (Figure 2 D)?

### Detailed Visualizations at Protein Level

After uploading the input file, the user can select a protein for a detailed analysis from a table, or directly search for a protein via its UniProt^39^ name and accession. The selected protein can then be viewed in two different ways: The modification view shows the distribution of modification sites across the amino acid sequence, while the structure view shows the modifications in a 3D structure context.

#### Modification View

**Figure 3:**
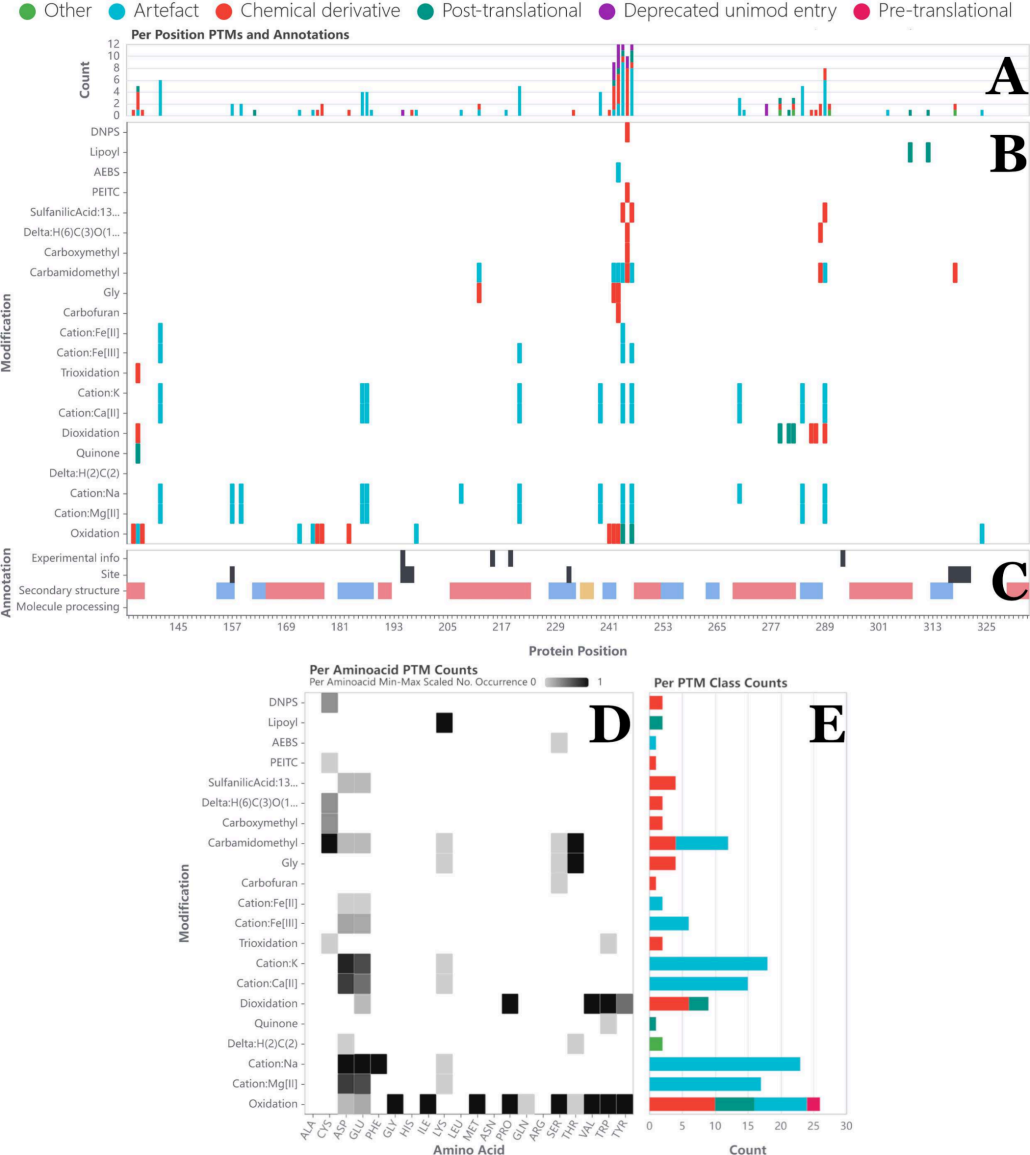
*Single protein modification visualizations of PTMVision*. (A): Bar chart with the modification count per position; color indicates UniMod class. (B): Presence/absence plot, where each row represents a modification, and each column a position in the protein sequence. A filled cell indicates that the modification type was found at the respective position. Modifications are sorted by their mass shift and color indicates UniMod class. (C): Tracks below the presence/absence plot show information mapped from UniProt (e.g., domains, binding sites, and known modification sites). (D): Heatmap showing the distribution of modification types across amino acids. Values are normalized per amino acid. (E): Bar chart showing the number of sites per modification type (note that a modification can be of a different class depending on the modified amino acid). (A), (B), and (C) have a shared *x*-axis, (B), (D), and (E) share a *y*-axis.

The modification view offers a comprehensive overview of the types of modifications detected in the selected protein and where the sites are located on the primary sequence. The presence/absence matrix shows the sites in detail for each modification type. Above it, the stacked bar chart summarizes how many different PTM types were identified per site, while the site counts per modification type are shown in the bar chart to the far right. Context for the matrix is provided by tracks below that show the protein annotations retrieved from UniProt.^39^ A heatmap shows normalized counts of modifications across amino acids. This normalization is performed for each amino acid to allow prevalent modifications for a given amino acid to be easily spotted. The modification view can be used to find positions that carry many different modifications, to cross reference identified modifications with those already annotated in UniProt, and to see which modification types have the most sites on the protein. It is also possible to use this view for identification quality control. For instance, if different modifications with highly similar mass shifts are found on the same residue, this might be due to incorrect classification by the search engine. As the presence/absence map is sorted by induced mass shift, potential misclassifications are directly identifiable in the modification view as vertical stripes. Horizontal stripes can indicate unreliable PTM localizations, or PTM hotspots. The different subplots are all linked to each other via shared axes and zooming behavior. For instance, zooming onto a domain of interest in a UniProt track (Figure 3 C) will also zoom the histogram and the presence/absence map to that region.

#### Structure View

**Figure 4:**
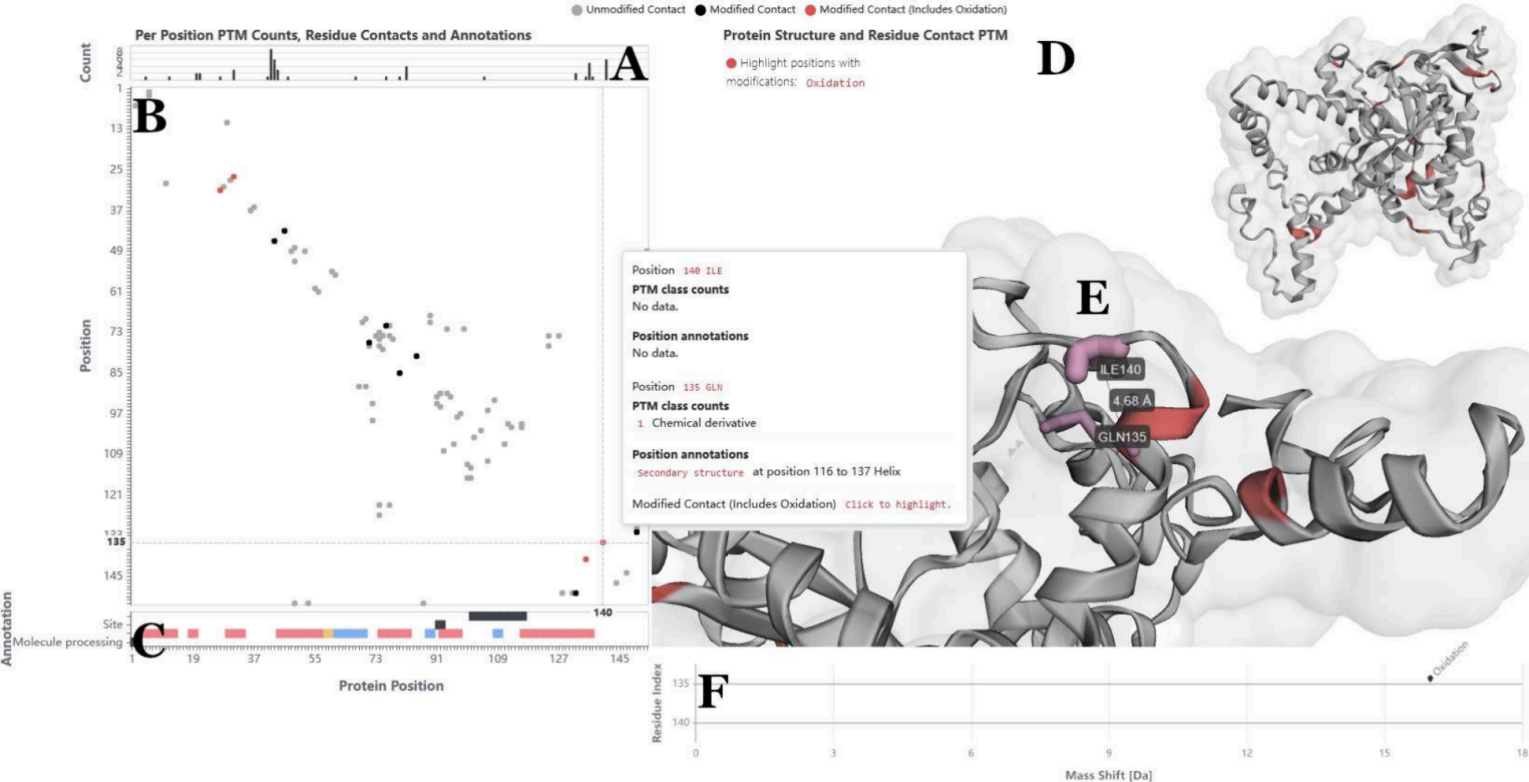
*Single protein structure and contact visualizations of PTMVision*. (A): Bar chart with the modification type count per position. (B): Contact map showing residues in close proximity. A cell is colored if the respective residues are in close contact, here defined as Cβ (Cα for glycine) distance <4.69 Å. Color indicates the modification state of the respective residue pair: Light gray cells represent pairs of amino acids that are in contact, with both residues unmodified. Dark gray cells represent pairs of amino acids that are in contact, with at least one of the residues modified. If a modification was highlighted by the user (here oxidation), residues that are in contact and where one or both carry the selected modification are colored in red. (C): Tracks showing the annotations retrieved from UniProt. (D): 3D Structure from the AlphaFold Protein Structure Database. Sites that carry the selected modification will be highlighted on the structure as well. (E) Selecting a residue pair (i.e., a cell in the contact map) will highlight the respective residues in the structure model. (F): Modifications of the selected residue pair, plotted according to their mass shift.

The second view focuses on structure, as the placement of modifications on the protein structure can be key to understand their potential functional implications. PTMs can have wide-ranging effects through the alteration of interactions between residues in close contact, for example by steric hindrance, or by masking other PTM sites.^46^ The exploration of such potential interactions is enabled via a contact map (see Figure 4 A) that is linked to the 3D protein structure (see Figure 4 D). Protein contact maps are two-dimensional representations of three-dimensional protein structures, in which the axes correspond to the positions in the primary sequence, and filled cells indicate that these two residues are in close contact, i.e., their Cβ (or Cα for glycine) atoms are close to each other in 3D space. As a distance cutoff we chose 4.69 Å, a value previously reported in the literature as an estimation for physical interaction of PTMs based on an empirical analysis.^47^ Potential PTM–PTM or PTM-residue interactions are indicated by black cells, and if a modification was selected for highlighting, possible interactions where one or both residues carry this modification are highlighted in red, making these readily identifiable. Zooming is linked between the contact map, the annotation tracks, and the histogram. The 3D structure is interactive as well, and selecting a cell in the contact map will highlight the respective residues in the structure.

### Example Applications

Here we look at the application of PTMVision to two data sets in detail to demonstrate its capabilities: First, a human phosphoenriched data set to show how it can be used for basic quality control in the context of enrichment protocols, and second, a nonenriched *Escherichia coli* sample to demonstrate how PTMVision can be used to develop biological hypotheses.

#### Phospho-enriched Human Data Set

In the first use case, PTMVision was employed to analyze open search results from an LC-MS/MS run of a human colorectal cancer sample enriched for phosphorylated peptides using Ti^4+^-IMAC (PRIDE project PXD007740, run GEF_6h_R1_IMAC_2).^27^ The obtained data was reprocessed using FragPipe/MSFragger^7,32,43−45^ with carbamidomethylation of cysteine, oxidation of methionine, and phosphorylation of serine, threonine, and tyrosine as variable modifications, setting the fragment mass tolerance to 20 ppm. The precursor mass window was kept at the default open search value of −150 to 500 Da. The UniProt^39^ human reference proteome (UP000005640, downloaded on June 26, 2024) served as the database, concatenated with known contaminants. Search results were filtered at 1% protein FDR level and mass shifts were characterized with PTMShepherd.^45^

**Figure 5:**
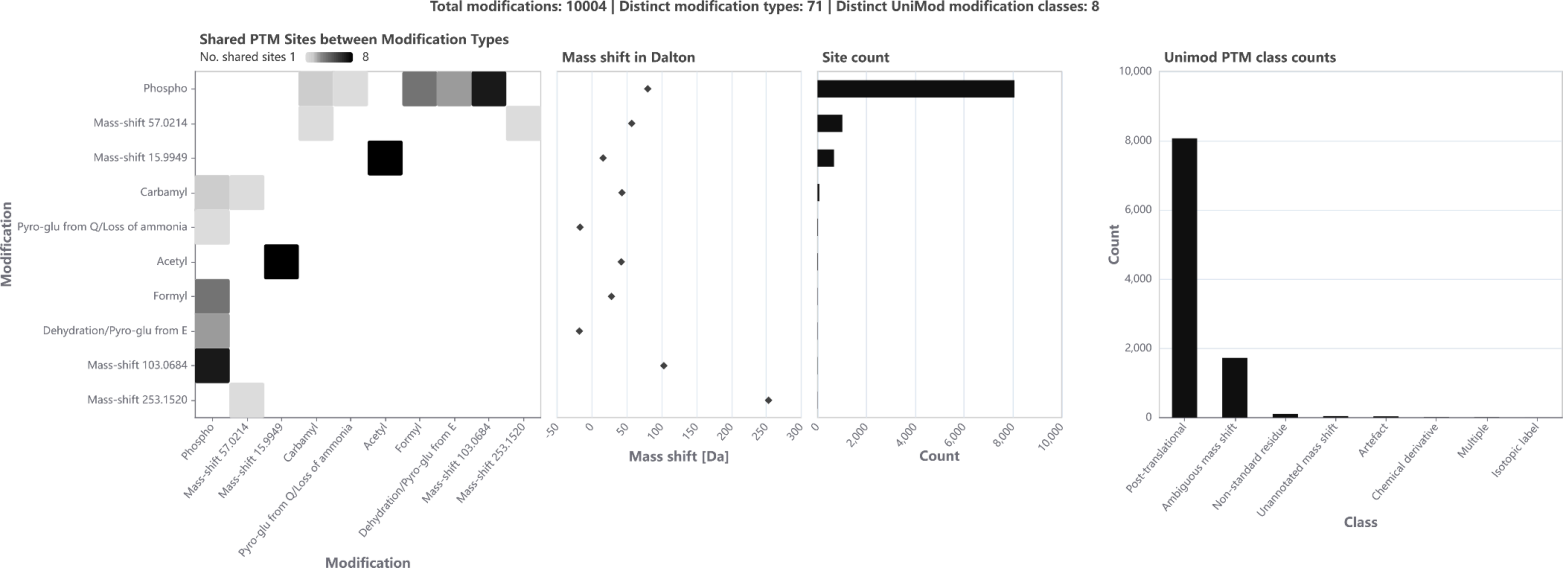
*PTMVision overview panel from the phospho-enriched data set PXD007740; Application screenshot*. The modification types are sorted by number of sites, the top 10 most common types are shown. Besides the enriched modification, 70 other modification types were localized but on substantially less sites. Phosphorylation shares the most sites with a mass shift of Δ103.0683 Da that could not be annotated with a UniMod modification. For the PTM Class counts, these mass shifts are grouped under “Ambiguous mass shift”.

The PTMVision overview indicates that phosphorylation was the predominant modification in the enriched sample (Figure 5), with 8068 phosphorylation sites identified across 94% of all detected proteins. Depending on the user defined mass resolution, PTMVision suggests that similar mass shifts may result from sulfonation (Δ0.009516 Da), which could potentially be coenriched due to the chemical similarity of the modifications. Other identified but ambiguously matching mass shifts include Δ57.0214 Da (most likely carbamidomethyl, matching to carbofuran, A → Q, G → N substitutions, or addition of G as well) and Δ15.9949 Da (most likely oxidation, matching to A → S and F → Y substitutions as well). Pyrophosphorylation, a modification that could be enriched by IMAC as well, is only localized on two sites. An unannotated mass shift of Δ103.0684 Da co-occurred with phosphorylation at all of its seven identified sites, suggesting a potential combination of phosphorylation with another molecule.

#### Unenriched *Escherichia coli* Data Set

Additionally, PTMVision was used to analyze the open search results of an LC-MS/MS run of an unenriched *E. coli* sample (PRIDE project PXD000498, run A14–07122),^26^ reprocessed using ionbot^10^ version 0.11.3 with carbamidomethylation of cysteine and oxidation of methionine as variable modifications. Precursor and fragment mass tolerances were set to 10 ppm and 0.02 Da, respectively, and the UniProt *E. coli* reference proteome (UP000000625, downloaded on June 27, 2024) served as the database. Search results were filtered at 1% PSM level FDR.

**Figure 6:**
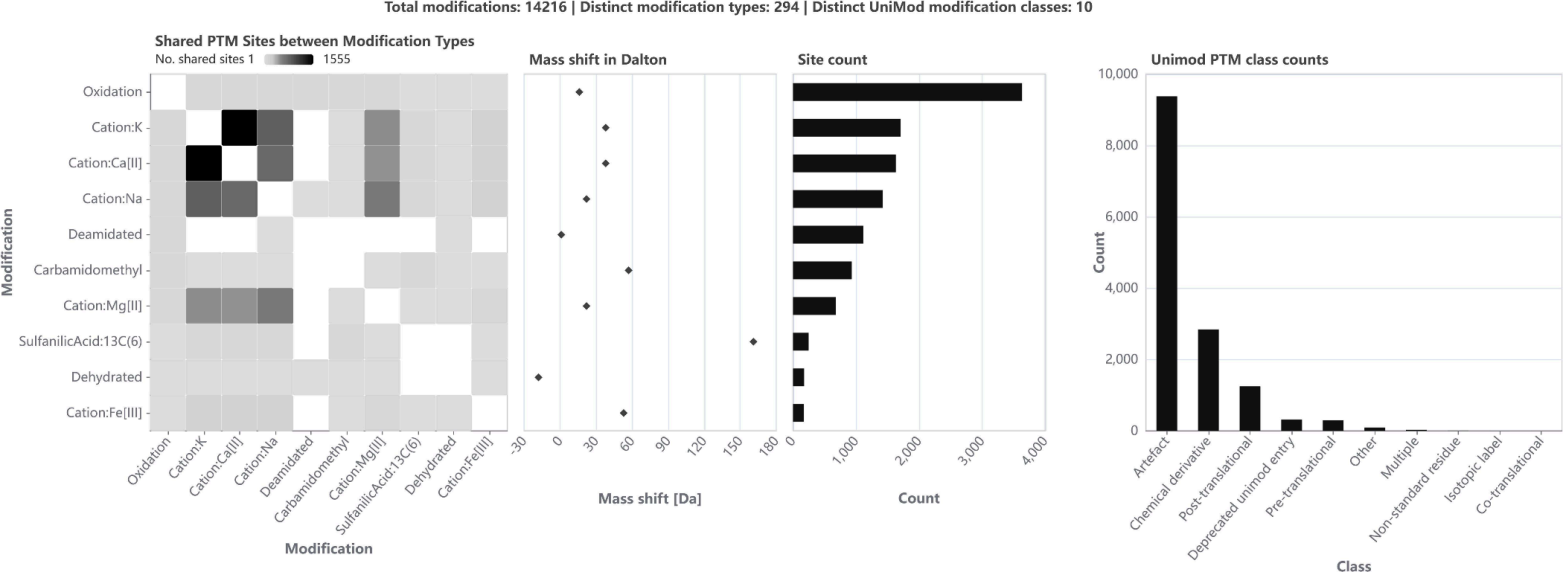
*PTMVision overview panel from the nonenrichedEscherichia colidata set PXD000498; Application screenshot*. The panel shows a summary of the PTMs and modification sites of the sample, with the top 10 most common modification types shown.

The overview panel provides a way to get an intuition about the sample-wide PTM site landscape (Figure 6, zoomed in on the top ten modification types). The first thing that stands out is the high number of different modifications and types identified, with 14,216 localized modification events with 294 different types. A few modification types are highly abundant, after that the distribution quickly drops off until reaching a long tail of modification types with less than 20 localized sites that might stem from false positive PSMs. The search identified both sites for commonly studied bacterial modifications such as phosphorylation (32 sites), acetylation (24 sites), and oxidation (3,634 sites), as well as noncanonical modifications like cation adducts (e.g., calcium: 1,631 sites, potassium: 1,707 sites, sodium: 1,423 sites). A substantial overlap of modification sites of cation adducts is visible in the shared sites heatmap. The shared sites between potassium and calcium adducts might be artifacts from wrong mass shift assignments, as the mass difference between the two is only 0.01 Da and therefore potentially below the fragment mass resolution. However, the mass shift induced by sodium adducts is clearly distinguishable from the other two, while sharing a substantial number of sites with the other cation adducts (757 sites with calcium, 800 with potassium). These modifications could be further investigated, as well as the extremely high mass shifts visible in the scatter plot. Focusing on or filtering out specific modification classes such as the 9,380 modifications assigned to be artifacts might also yield interesting insights.

**Figure 7:**
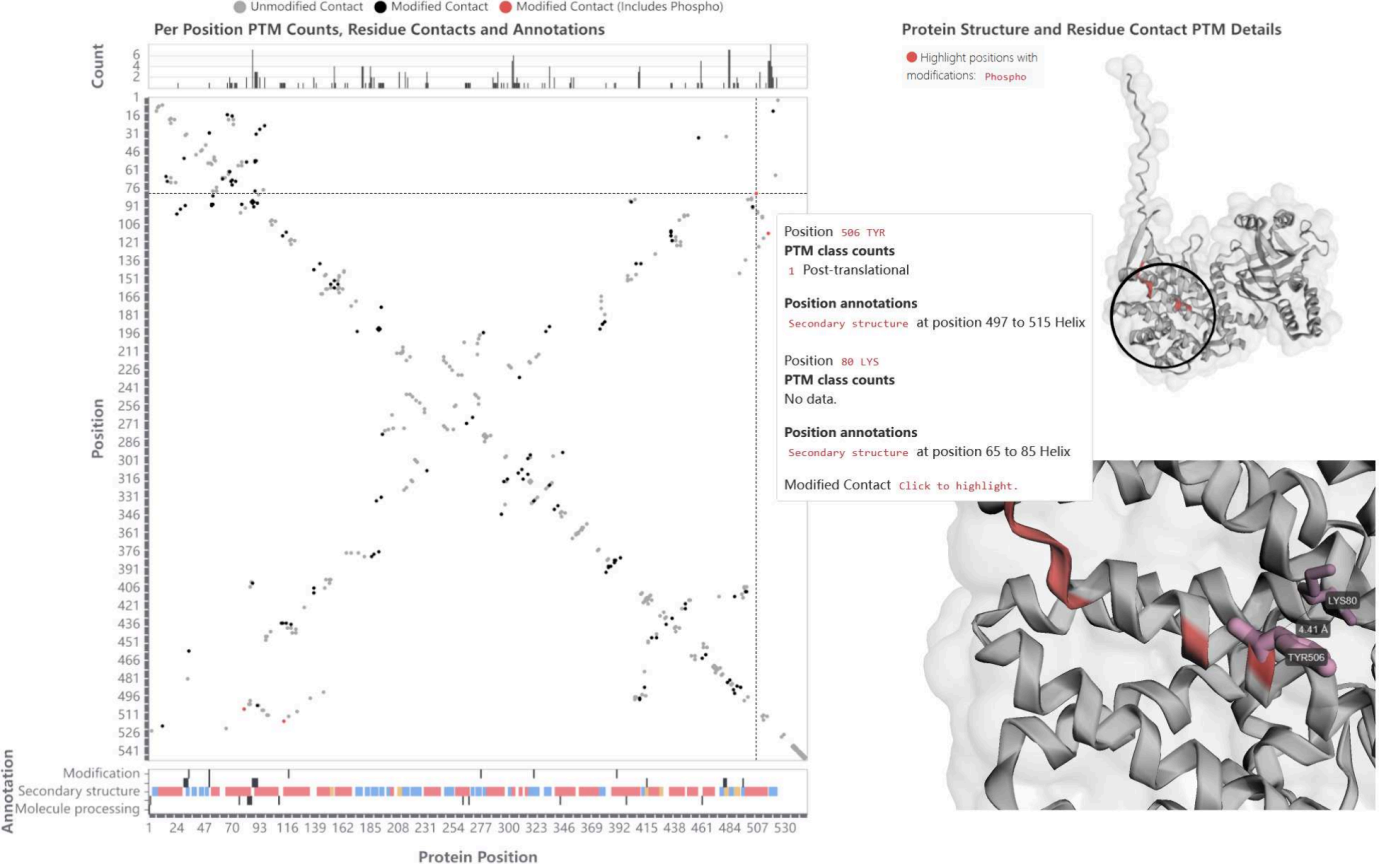
*PTMVision structure view of GroEL (CH60*_*ECOLI)*. (**Left**) Amino acid contacts without modifications are filtered in the contact map, and contacts including one or two phosphosites are highlighted in red. The residue pair at positions 506 and 80 is highlighted. (**Right**) The zoomed in 3D structure (bottom, cf. encircled part in the entire structure above) shows the distance to the neighboring residue Lys80 of the phosphosite Tyr506 in Å. Due to a loop, Lys80 is in proximity and could interact with the negatively charged phosphate group.

For more specific protein-level analyses, the user can select a protein of interest from the protein table and investigate its PTMs in detail. Figure 7 shows the structure view for chaperonin GroEL of *E. coli*. GroEL facilitates the proper folding of other proteins, which is particularly critical during heat stress, as elevated temperatures can cause proteins to denature and require refolding. Heat shock-induced phosphorylation of GroEL has been reported to enhance its binding capacity to several denatured proteins.^48^ The underlying mechanism for this increased binding affinity remains unknown.

When using the highlighting function to bring out the identified phosphosites in the structure view, a residue contact between Lys80 and pTyr506 becomes prominent. Clicking on the Lys80-pTyr506 cell in the contact map shows the two amino acids in the 3D structure (Figure 7, right side). From the structure, it becomes clear that Lys80 is 4.41 Å away from pTyr506, leading to the hypothesis that the positively charged lysine interacts with the negatively charged phosphogroup of Tyr506. This interaction could change the conformation to a more binding-competent state, for example by making the binding sites more accessible.

#### Additional Example Applications

The application of PTMVision to all compiled data sets described in the Materials and Methods section are provided as example sessions on the application’s Web site to demonstrate the use of the supported data formats without a dedicated data upload by users—however, with the exception of the two described before, the analysis results were not extensively examined and primarily serve as a proof of concept for the support of the respective data formats. Details of the available examples are given in Supplementary Table S1 and on the PTMVision Web site.

## Conclusions

The PTMVision web server is an intuitive and easily accessible tool that facilitates rapid assessments as well as in-depth analysis of the modification landscape of proteins. It enables reseachers to generate hypotheses by visually exploring modifications identified at different levels and from different viewpoints. Due to their interactive nature, the visualizations scale to larger open search results and remain comprehensive and easy to navigate. The modification view shows the modification site distribution across the primary structure in an uncluttered fashion, with more details on demand to facilitate both broader and more specific analyses. A new aspect is the incorporation of a contact map that underscores the structural context of modifications, providing insights into their potential interactions with the protein’s structural environment. By combining and linking the contact map with the predicted 3D model, the structure view nicely complements the sequence-based modification view. In the future, we plan to extend PTMVision with features developed during past workshop discussions: Including PSM level information to show sequence coverage, providing direct links to the original spectra for manual validation, and adding visualizations that compare the modification landscapes between two samples.

## Data Availability

PTMVision is available at https://ptmvision-tuevis.cs.uni-tuebingen.de. The source code of PTMVision—including a description on how to run PTMVision locally via Docker—is hosted on GitHub at https://github.com/Integrative-Transcriptomics/PTMVision. The following resources are hosted on Zenodo at 10.5281/zenodo.13270568: ptmvision-code.zip: The source code of PTMVision at the time of publication of this manuscript. ptmvision-image.tar.gz: The Docker image of PTMVision at the time of publication of this manuscript. ptmvision-supplementary-table-1.xlsx: Description of the raw input files origin and processing procedure/methods for the use cases, i.e., the example applications in this manuscript and available on the web server. ptmvision-usecases.zip: The sequences (in FASTA format), search engine outputs, and PTMVision session files for the two example applications described in detail in this manuscript. The raw input files are not included. ptmvision-examples.zip: The input and PTMVision session files for the example sessions available on the web server. The raw input files are not included.

## Supporting Information Available

Table S1: Details on the origin of raw data and search result files for example applications/sessions in PTMVision (XLSX)
